# Development and validation of bile acid profile-based scoring system for identification of biliary atresia: a prospective study

**DOI:** 10.1186/s12887-020-02169-8

**Published:** 2020-05-27

**Authors:** Dongying Zhao, Kejun Zhou, Yan Chen, Wei Xie, Yongjun Zhang

**Affiliations:** 1 Department of Neonatology, Xinhua Hospital, Shanghai Jiao Tong University School of Medicine, 1665 Kong Jiang Road, Shanghai, 200092 China; 2 Department of Pediatric Surgery, Xinhua Hospital, Shanghai Jiao Tong University School of Medicine, Shanghai, China; 3 Department of Pediatric Surgery Intensive Care Unit, Xinhua Hospital, Shanghai Jiao Tong University School of Medicine, Shanghai, China

**Keywords:** Bile acid, Biliary atresia, Infantile cholestasis, Scoring system

## Abstract

**Background:**

Early distinguishing biliary atresia from other causes of infantile cholestasis remains a major challenge. We aimed to develop and validate a scoring system based on bile acid for identification of biliary atresia.

**Methods:**

In a prospective study, a total of 141 infants with cholestasis were enrolled in two sets (derivation cohort, *n* = 66; validation cohort, *n* = 75) from 2014 to 2018. Variables with significant difference between biliary atresia and non-biliary atresia infants were selected in the derivation cohort. Then, a scoring system including those variables was designed and validated.

**Results:**

Among 66 patients in the derivation cohort, 34 (51.5%) had biliary atresia. A scoring system was proposed with the following variables: glycochenodeoxycholic acid/chenodeoxycholic acid, clay stool, and gamma-glutamyl transferase. The total score ranged from 0 to 41, and a cutoff value of 15 identified biliary atresia with an area under receiver operating characteristic curve of 0.87 (95% confidence interval, 0.77–0.94), sensitivity of 85.3%, and specificity of 81.3% in the derivation cohort; these values were also confirmed in a validation cohort with a sensitivity of 90.0% and specificity of 80.0%.

**Conclusions:**

The proposed simple scoring system had good diagnostic accuracy for estimating the risk of biliary atresia in infants with cholestasis.

## Background

Biliary atresia (BA) is a life-threatening disease, and its survival and prognosis correlate directly with early diagnosis and timely surgical treatment (Kasai procedure) [1, 2]. However, identifying BA from other infantile cholestasis at the early stage of the disease is still challenging because of similar clinical, biochemical, imaging, and histopathological characteristics [3, 4]. Intraoperative cholangiography (IOC) may clearly reveal the biliary tree, supporting the diagnosis of BA, but it is an invasive and inconvenient procedure and could considerably increase morbidity [5]. Stool color card has been commonly used as noninvasive method for region-wide screening BA with a high sensitivity, [6, 7] but it showed a mild-to-moderate specificity for differentiating BA from non-BA cholestasis [8]. Therefore, it is essential to develop a preoperative, non-invasive, and practical investigation for the diagnosis of BA in infants with cholestasis.

Clinical assays for detecting bile acid profiles might be feasible non-invasive biomarkers [9]. Several studies have reported altered serum bile acid profiles, including intrahepatic cholestasis in pregnancy, [10] nonalcoholic fatty liver disease, [11] and neonatal intrahepatic cholestasis caused by citrin deficiency [12]. It was proposed that different types of serum bile acids could be found in circumstances where enterohepatic circulation of bile acids is obstructed. Other diagnostic methods, such as γ-glutamyl transferase (γ-GT) levels, abdominal ultrasonography (US), and hepatobiliary scintigraphy (HBS), may be helpful in BA diagnosis; however, the effectiveness of these methods remains unsatisfying, [5, 13] limiting the clinical application for identifying BA alone. We hypothesized that a combination of multiple clinically examined parameters may be a potential solution for identifying BA in infants with cholestasis.

In this study, we aimed to develop a scoring system using a prospective cohort by combining clinical characteristics and multiple biomarkers, including simultaneous bile acid assay, to differentiate BA from infantile cholestasis and to validate the potential diagnostic value of this system.

## Methods

### Participants and study design

This was a prospective study including two consecutive cohorts of infants with and without cholestasis, which was approved by the ethical committee of Xinhua Hospital, Shanghai Jiaotong University School of Medicine. Parents or legal guardians signed written informed consent for participation. The enrollment period was from June 2014 to May 2016 (derivation cohort) and from June 2016 to June 2018 (validation cohort). The inclusion criteria were as follows: 1) conjugated bilirubin > 20% of the total bilirubin when the total bilirubin was ≥85 μmol/L and ≥ 17 μmol/L when the total bilirubin was < 85 μmol/L [14]; 2) age at first visit ≤90 days; and 3) gestational age ≥ 34 weeks. During the study period, we also enrolled inpatients who had pneumonia but with normal liver function and without congenital malformation in the same age and gestational age range as controls, in order to obtain a standard reference value of individual bile acid (IBA) concentrations.

Upon admission of cholestatic infants to the neonatal department or pediatric surgery ward of our hospital, a relatively rapid series of investigations were conducted to establish the etiologies. In this study, 1 ml of initial serum samples was collected for detecting serum bile acid profiles to derive the biomarker-based formula to discriminate infants with and without BA. Management of cholestatic infants included history taking and physical examination, measurements of liver function panels, IgM and IgG of Cytomegalovirus and Epstein-Barr virus, hepatitis B surface antigen, US, and HBS. Acylcarnitines and amino acid profiles in dry blood spot and organic acid profiles in urine were also detected to establish the diagnosis of metabolic disorders. For infants suspected of congenital disorders, genetic testing was performed. If BA could not be ruled out by the aforementioned investigations, IOC and liver biopsy were done for the suspect infants. All infants had 1–3 months of clinical follow-up. The exclusion criteria were as follows: metabolic cholestasis such as neonatal intrahepatic cholestasis caused by citrin deficiency, choledochal cysts, and severe malformations in other systems.

Finally, 141 infants were assigned to one of two groups: BA group (*n* = 74) and non-BA group (*n* = 67). The diagnosis of BA was confirmed based on IOC findings that revealed an obstruction of bile duct in combination with histological features of liver biopsies [15, 16]. Infants were assigned to the non-BA group if they met either of the following criteria: 1) cholangiography showing a patent biliary tree, 2) recovery from cholestasis and normalized laboratory values during the clinical follow-up period. The ultimate diagnosis of non-BA included idiopathic neonatal hepatitis (*n* = 28), cytomegalovirus hepatitis (*n* = 27), parenteral nutrition associated cholestasis (*n* = 9), sepsis (*n* = 2), and Alagille’s syndrome (*n* = 1). Additional 37 gestational age- and age-at-test-matched controls were also enrolled.

Demographic and clinical data were collected. Abnormal gallbladder was defined according to US findings as non-visualization of the gallbladder or gallbladder length ≤ 15 mm [17]. The presence of the triangular cord sign and internal diameter of the common hepatic duct were also evaluated by US. A positive HBS was defined as the absence of radiotracer in the intestines up to 24 h [17].

### Serum bile acid profile measurements

Serum samples were collected in 1.5 ml Eppendorf tubes and stored at − 80 °C until analyzed centrally at the Instrumental Analysis Center of Shanghai Jiaotong University (Shanghai, China). Fifteen IBA concentrations were determined using liquid chromatography-tandem mass spectrometry (LC-MS/MS) on the ACQUITY UPLC system (Waters, USA) coupled with SCIEX SelexION Triple Quad 5500 System (ABI-SCIEX, USA). Bile acid standards including ursodeoxycholic acid, glycocholic acid (GCA), deoxycholic acid, cholic acid (CA), tauroursodeoxycholic acid, chenodeoxycholic acid (CDCA), lithocholylglycine acid, glycoursodeoxycholic acid, lithocholic acid, taurolithocholic acid, glycochenodeoxycholic acid (GCDCA), glycodeoxycholic acid (GDCA), taurochenodeoxycholic acid (TCDCA), taurocholic acid (TCA), and taurodeoxycholic acid were purchased from Sigma-Aldrich (St. Louis, USA).

For the sample preparation, 50 μL of each serum sample was added with 200 μL of methanol/acetonitrile (5:3), vortexed briefly to mix, and then incubated for 30 min at 4 °C. After centrifugation at 16,000 g for 15 min, all the supernatant was transferred to a clean tube and was dried under a gentle stream of nitrogen at room temperature. The residues were reconstituted with 200 μL of 50% methanol aqueous for LC-MS/MS analysis. The process for the determination of IBAs was similar to those previously reported with slight modifications [9, 18, 19]. Data analysis was performed with Analyst Software 1.5.2 (Applied Biosystems, USA). Bile acid concentrations of each sample were finally exported to Excel spreadsheets.

### Statistical analysis

Statistical analysis was performed using SAS 9.2 statistical software version (SAS Institute, Inc., Cary, North Carolina), and illustrations were plotted using Origin 9 (OriginLab Corp., Northampton, Massachusetts). Descriptive results were expressed as mean ± standard deviation (SD), median (interquartile range, IQR), or number (percentage) of individuals with a condition. Chi-square test or Fisher’s exact test was used for categorical variables, and ANOVA analysis or Kruskal-Wallis test for continuous variables whenever necessary. A *p* value of < 0.05 in those method was considered significant. Paired comparisons among three groups were using Mann-Whitney test and a p value < 0.017 was considered significant. A prediction model was thereafter constructed by stepwise selection of multivariate logistic regression analysis of assessment factors determined to be statistically significant in the univariate analysis.

The diagnostic performances of the individual variables as well as combination of variables were expressed by a receiver operating characteristic (ROC) curve. A scoring system was thereafter derived on the basis of the coefficients of the predictors in the final multivariable model using the model developed by Sullivan et al., [20] in which points were assigned to each variable with point totals corresponding to risk estimate. High- and low-risk cutoff points for the BA risk score were determined by the cutoff in the derivation phase.

## Results

### Demographics and clinical data

A total of 66 patients from the derivation cohort, 75 patients from the validation cohort, and 37 age-matched controls were enrolled. The mean age was 50.2 ± 14.6 (median: 52) days and 55.3 ± 16.9 (median: 58) days for the derivation cohort and validation cohort, respectively. There were no significant differences in birth weight (3134.4 ± 618.9 g vs 2984.0 ± 502.2 g, *p* > 0.05) and weight at admission (4254.8 ± 872.2 g vs 4466.6 ± 1112.3 g, *p* > 0.05) between the two cohorts. Overall, 63 female and 78 male cholestatic infants were enrolled. A total of 105 infants underwent intraoperative cholangiography and liver biopsy, of which 74 BA cases and 31 non-BA cases were identified. Another 36 infants were assumed to have no BA due to the recovery of cholestasis during the clinical follow-up.

Baseline patient characteristics of the derivation cohort are shown in Table 1. Demographic and clinical parameters including birth weight, age and weight at admission, sex, parity, recurrent jaundice, and splenomegaly showed no significant differences between the BA and non-BA groups (*p* > 0.05, all). The frequency of clay stool and hepatomegaly was higher in the BA group than in the non-BA group. There was no apparent significant difference in liver function tests except for total bilirubin and γ-GT between the BA and non-BA group. γ-GT levels were much higher in the BA group than in the non-BA group (*p* < 0.001). The frequency of abnormal gallbladder size and positive findings on hepatobiliary scintigraphy were also significantly higher in the BA group than in the non-BA group.

**Table 1:** Clinical, laboratory, ultrasonographic, hepatobiliary scintigraphy characteristics of the derivation cohort

Parameters	BA	Non-BA	Control	***P***	BA vs Non-BA
	*n* = 34	*n* = 32	*n* = 37		***P***^a^
**Baseline characters**					
Maternal age (year) (Mean, SD)	30 (4)	26 (3)	30 (4)	0.437	0.272
Birth weight (g) (Mean, SD)	3232 (461)	2931 (770)	3393 (477)	0.241	0.218
Age at admission (day) (Mean, SD)	51 (16)	50 (13)	42 (12)	0.151	0.176
Weight at admission (g) (Mean, SD)	4514 (844)	3951 (818)	4264 (1014)	0.306	0.096
Parity (N, %)				0.013	0.981
0	24 (70.6)	23 (71.9)	28 (75.7)		
≥ 1	10 (29.4)	9 (28.1)	9 (24.3)		
Sex (N, %)				0.117	0.051
Male	13 (38.2)	20 (62.5)	16 (43.2)		
Female	21 (61.8)	12 (37.5)	21 (56.8)		
**Clinical measures**					
Recurrent jaundice (N, %)	4 (11.8)	9 (28.1)	/	/	0.097
Clay stool (N, %)	23 (67.7)	7 (21.9)	/	/	***< 0.001***
Hepatomegaly (N, %)	19 (55.9)	9 (28.1)	/	/	***0.024***
Splenomegaly (N, %)	7 (20.6)	7 (21.9)	/	/	0.899
**Liver function test (Median, IQR)**					
Total bile acid (μmol/L)	105 (73, 132)	82 (68, 124.5)	8 (6, 15)	***< 0.001***	0.141
Alanine transaminase (U/L)	111.5 (72, 235)	118.5 (87.5, 184)	40 (30, 50)	***< 0.001***	0.963
Aspartate transaminase (U/L)	219 (133, 308)	170 (132.5, 296.5)	50 (39, 61)	***< 0.001***	0.572
Alkaline phosphatase (U/L)	541.5 (446, 619)	510.5 (373, 618.5)	213 (171, 246)	***< 0.001***	0.959
Total bilirubin (μmol/L)	162.5 (143, 194)	141.5 (101, 220.5)	24 (16, 47)	***< 0.001***	***0.047***
Direct bilirubin (μmol/L)	107 (79, 124)	96 (49, 131)	0 (0, 0)	***< 0.001***	0.342
Albumin (g/L)	39 (35, 41)	36 (34, 40)	38 (34, 39)	0.153	0.091
γ-glutamyl transferase (U/L)	440 (222, 731)	117 (85, 273)	67 (41, 94)	***< 0.001***	***< 0.001***
**Ultrasonography findings**					
Abnormal gallbladder (N, %)	14 (41.2)	3 (9.4)	/	/	***0.003***
Triangular cord sign (N, %)	3 (8.8)	2 (6.3)	/	/	0.693
Internal diameter of common hepatic duct(mm) (Median, IQR)	1.2 (0.6, 1.5)	1.3 (1.0, 1.4)	/	/	0.712
**Positive finding in hepatobiliary scintigraphy** (N, %)	29 (85.3)	14 (43.8)	/	/	***< 0.001***

*BA* Biliary atresia

a Variance analysis, Mann-Whitney test or Chisq test of BA and non-BA group

### Serum bile acid concentration in BA, non-BA, and normal controls

Among the 15 IBAs, seven bile acids could be quantitatively detected in all infants (Table 2). Compared to controls, levels of CA and CDCA were significantly lower, while levels of GCA, GCDCA, TCA, and TCDCA were significantly higher in BA and non-BA infants. Differences in IBAs were also found between BA and non-BA infants. CDCA levels were significantly lower in the BA group, while GCA and GCDCA levels were significantly higher in the BA group than in the non-BA group.

**Table 2:** Bile acid assay of derivation cohort and normal controls

	BA (*n* = 34)	Non-BA (*n* = 32)	Control (*n* = 37)	***P***^a^
CA (ng/ml)	9 (8, 13)^c^	10.3 (8, 15)^d^	97 (60, 172)	***< 0.001***
CDCA (ng/ml)	16 (1, 21) ^b, c^	26 (14, 37)^d^	216 (108, 324)	***< 0.001***
GDCA (ng/ml)	5.2 (2.0, 7.2)^c^	1.8 (2.0, 5.9)	0.9 (0.5, 2.9)	***< 0.001***
GCA (ng/ml)	6160 (3476, 9978) ^b, c^	3044 (1274, 5920)^d^	820 (428, 1824)	***< 0.001***
GCDCA (ng/ml)	11,440 (7800, 18,600)^b, c^	5680 (3474, 10,960)^d^	2527 (1768, 3980)	***< 0.001***
TCA (ng/ml)	7936 (4740, 12,245)^c^	6080 (4080, 10,170)^d^	574 (249, 1684)	***< 0.001***
TCDCA (ng/ml)	16,160 (8720, 23,908)^c^	13,680 (8740, 19,600)^d^	1633 (784, 3892)	***< 0.001***
GCDCA/CDCA	685 (394, 1288)^b,c^	266 (100, 596)^d^	13 (6, 20)	***< 0.001***

Data are presented as median (IQR)

a P value: data were analyzed using Kruskal-Wallis test

b Paired comparisons with significance (*p* < 0.017), using Mann-Whitney test between BA vs Non-BA

c Paired comparisons with significance (*p* < 0.017), using Mann-Whitney test between BA vs Control

d Paired comparisons with significance (*p* < 0.017), using Mann-Whitney test between Non-BA vs Control

*CA* Cholic acid; *CDCA* Chenodeoxycholic acid; *GDCA* Glycodeoxycholic acid; *GCA* Glycocholic acid; *GCDCA* Glycochenodeoxycholic acid; *TCA* Taurocholic acid; *TCDCA* taurochenodeoxycholic acid

GCDCA is generated by glycine conjugation of CDCA in the liver. Because there were higher GCDCA levels and lower CDCA levels in BA, we used the ratio of GCDCA/CDCA to compare BA infants with non-BA infants. The ratio of GCDCA/CDCA was significantly higher in BA infants than in non-BA infants (*p* < 0.05). The median ratio of GCDCA/CDCA was 685 (range,394–1288) in BA infants and was 266 (range, 100–596) in non-BA infants (Table 2).

### Derivation cohort

The variables that were statistically significant with *p* < 0.01 in the univariate analysis were included into the multivariate logistic regression analysis by stepwise selection. The final multivariable model included (1) γ-GT, (2) GCDCA/CDCA ratio, and (3) clay stool.

**Fig. 1:**
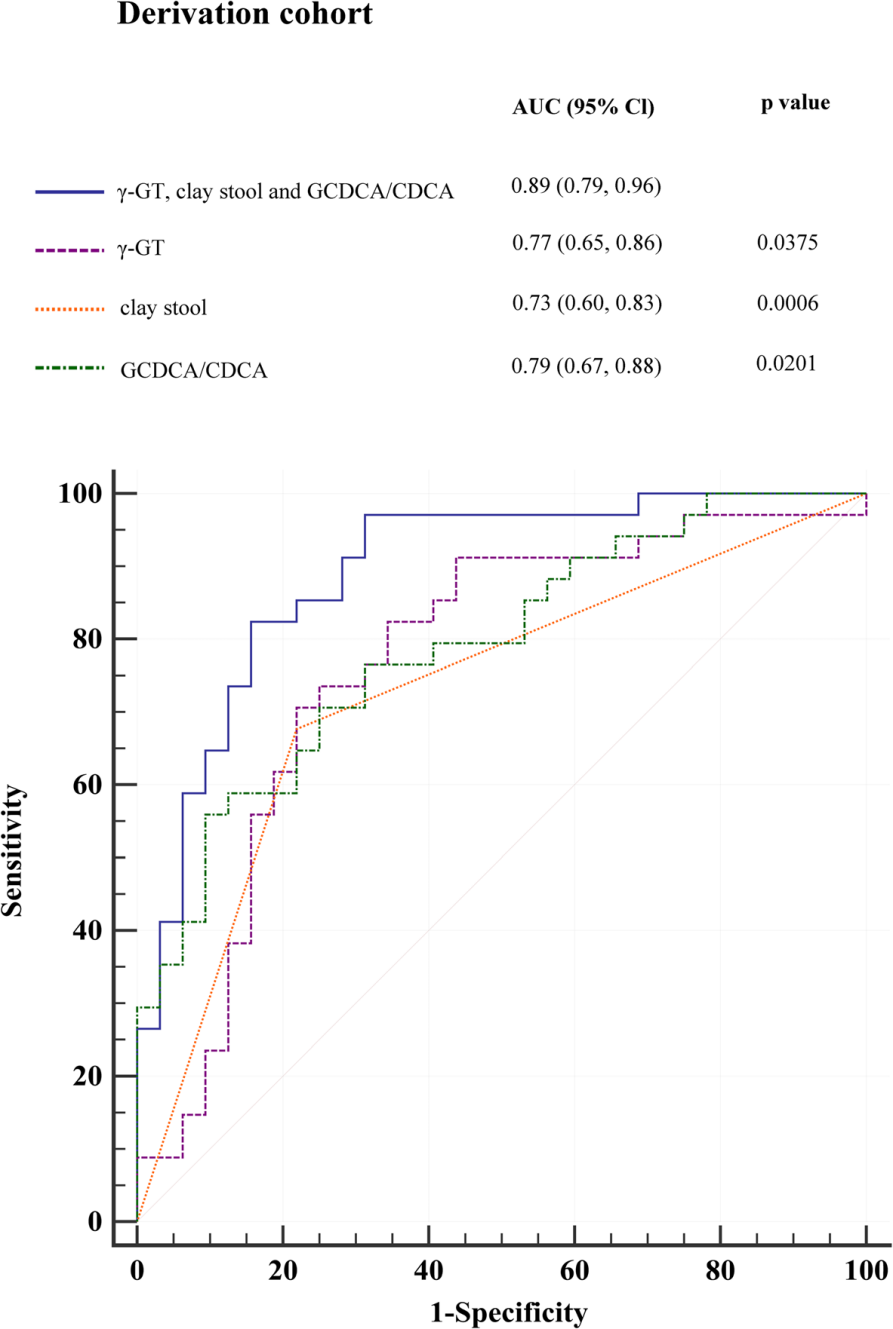
Diagnostic performance of γ-GT, clay stool, and GCDCA/CDCA compared to the combination of three variables in the derivation cohort.The areas under receiver operating characteristic curve (AUC) for γ-GT, clay stool, and GCDCA/CDCA were 0.77, 0.73, and 0.79, and for γ-GT, clay stool, and GCDCA/CDCA was 0.89. *P* values show the AUC for combination variables versus the AUC for each individual variable

In the derivation cohort, using a ROC curve, the diagnostic performance of the three selected variables based on the occurrence of BA was evaluated individually and compositely. A combination of these three parameters was proven to be significantly related to the identification of BA compared with each parameter (*p* < 0.05) (Fig. 1).

Accordingly, a formula with the three aforementioned variables was developed by stepwise algorithms for discriminating patients with BA from those with infantile cholestasis. The probability of BA = exp. (− 2.4672 + 0.1377 × γ-GT + 0.0319 × GCDCA/CDCA + 1.5779 × clay stool). It was obvious that the function is complicated and difficult for clinical application; therefore, a composite score system was then established for easy prediction (Table 3). Of note, because the IBA value varied depending on the instruments or reagents used, [12, 21] we standardized the serum bile acid value using the multiple of the median (MoM) value. Since we had tested the IBA concentrations of normal control, we could calculate the MoM, which is defined as the ratio of the actual measured value over the normal median value of IBA (Supplemental Table S1). Thus, the GCDCA/CDCA ratio could be practically used in any institution and hospital where serum bile acid profiles are measured. Similarly, the scoring system also contained MoM values of γ-GT to eliminate the different values in various laboratories. MoM values of GCDCA/CDDCA and γ-GT could replace the original value in the formula above, by which we could figure out the probability of BA.

**Table 3:** Points associated with selected predictor variables for multivariable model of BA in the derivation cohort

Predictor variable	Categories	Point^**a**^
γ-GT MoM	≤3 (ref)	0
	~ 6	3
	~ 9	5
	>9	10
GCDCA/CDCA MoM	≤5 (ref)	0
	~ 50	5
	100	15
	>100	30
Clay stool	0	0
	1	1

*MoM* Multiple of the median; *γ-GT* Gamma glutamyl transpeptidase; *CDCA* Chenodeoxycholic acid; *GCDCA* Glycochenodeoxycholic acid

a Points were assigned to each variable with point totals corresponding to risk estimate for BA

**Fig. 2:**
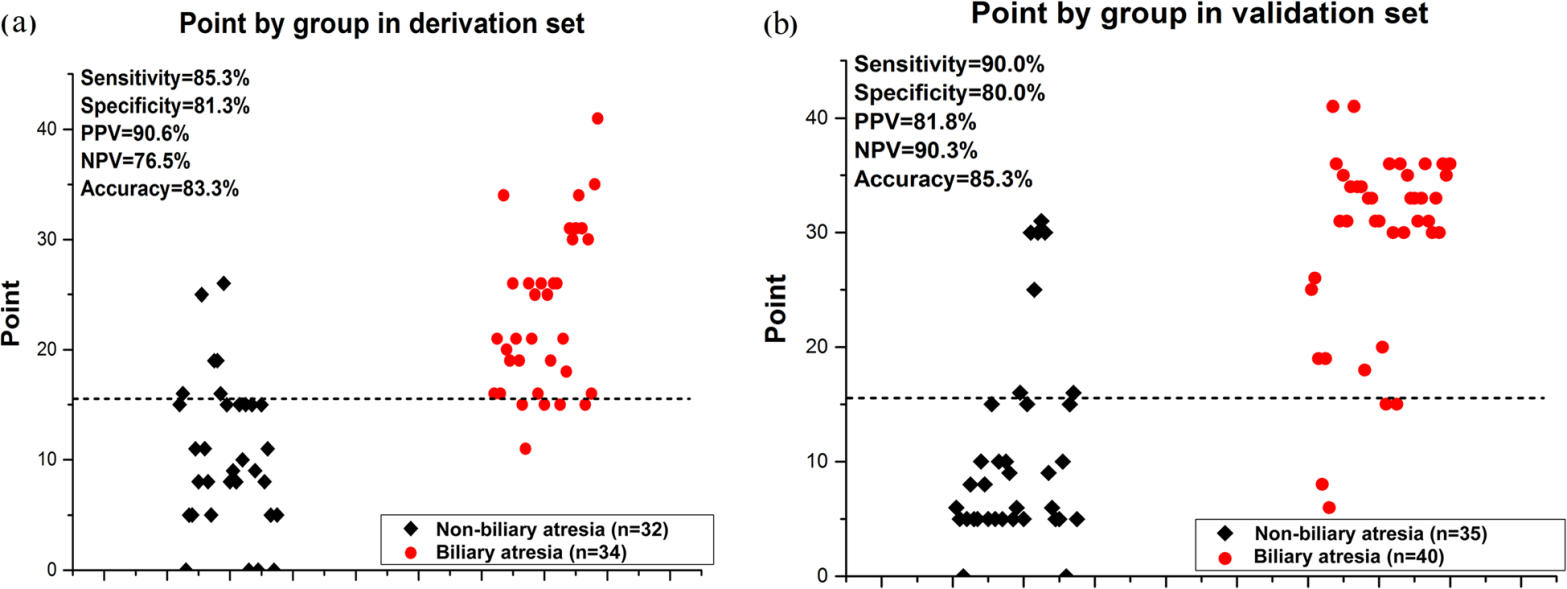
A three-variable score system in individual infants in the derivation cohort and validation cohort. The dashed line represents the score cutoff value of 15. The sensitivity and specificity rates to diagnose BA were 85.3 and 81.3% in the derivation cohort (**a**) and 90.0 and 80.0% in the validation cohort, respectively (**b**)

The BA score system derived from the multivariable model (score range, 0 to 41) linearly corresponded to the risk estimate. A ROC curve analysis was applied to evaluate the diagnostic efficacy of the score system. A cutoff point was selected to stratify BA risk (low risk, ≤15 points; high risk, > 15 points; Supplemental Table S2). The AUC of the scoring system was 0.87 (95% CI, 0.77–0.94). A scatter plot showed the diagnostic sensitivity of 85.3%, and specificity of 81.3% with a cutoff point of 15 (Fig. 2).

### Validation cohort

To verify the applicability of the proposed scoring system, a validation cohort of infants with cholestatic liver diseases was tested, including BA (*n* = 40) and non-BA (*n* = 35). The diagnostic sensitivity and specificity were 90.0 and 80.0%, respectively (Fig. 2).

The validation test characteristics for all point values are shown in Supplemental Table S3, in which the performance of the three-variable scoring system in individual infants was compared to the final confirmed diagnosis in the validation cohort with a cutoff point of 15. In the BA group, 4/40 (10%) infants were misdiagnosed, while 11 of all infants were misdiagnosed based on this scoring system, for an accuracy of 85.3%.

## Discussion

Currently, no single, non-invasive diagnostic technique appears to be clearly superior to differentiate BA from other causes of cholestasis in infants. In this prospective study, we developed a three-variable scoring system and corresponding risk estimate (including γ-GT, clay stool, and GCDCA/CDCA) that showed the best performance for identifying BA in cholestatic infants before age 90 days.

Of all features assessed in this study, GCDCA/CDCA ratio, γ-GT, and clay stool were selected from a multiple clinical assessment and serum biomarkers by stepwise multivariate logistic regression analysis. We first developed an algorithm model for the diagnosis of BA including all three features in the derivation cohort. The AUC of such combination was 0.89 (95% CI, 0.79–0.96), which showed good discrimination of BA. Nevertheless, this model is too complex, difficult to use, and requires computer assistance. We then optimized the score system by using a quantitative scale. The final score system also showed good diagnostic ability for BA according to AUC value of 0.87 (95% CI, 0.77–0.94), similar to the original algorithm model. This final scoring system was easily calculated based on available clinical and laboratory data. Meanwhile, the scoring system using a cutoff of 15 also proved to have good diagnostic performance in the validation cohort. Furthermore, our scoring system provided estimation for infants suspected of BA into two risk categories that cover a wide range of BA diagnoses with an approximately 13-fold range of risk (from 7.4% at 0 points to 98.2% at 41 points); this could be a better reference for clinicians. In the high-risk group, scores > 35 had an estimated risk of BA of > 95.5%, and all patients in the validation cohort with higher scores were finally diagnosed with BA. Given the very high risk of BA in patients with scores higher than this, prompt intraoperative cholangiography should be recommended.

The prognostic value of serum IBAs as a rapid, non-invasive, and inexpensive additional diagnostic tool for differentiating BA from non-BA has been recently investigated [18, 22]. Higher GCDCA and lower CDCA levels were found in BA infants than in non-BA infants in this study as well as in other studies [22, 23]. CDCA is the primary bile acid synthesized in human pericentral hepatocytes. It is also a hydrophilic bile acid, thought to provide a hepatoprotective function. In cirrhosis patients, CDCA levels decreased, suggesting an impaired protective effect [24]. According to Chen, liver fibrosis is one the best indicators of BA [25]. We speculated that CDCA levels were significantly lower in BA infants than in non-BA infants because of the more severe fibrosis or cirrhosis due to pathological changes. Moreover, GCDCA is generated by glycine conjugation of CDCA in normal liver, which is excreted to the intestine through bile flow. Because there was obstruction of bile drainage, GCDCA in the liver was significantly elevated and reabsorbed via alternative export systems at the hepatic sinusoidal membrane, possibly causing the high levels in serum; in addition, the lack of intestinal bacterial interaction with conjugated bile acids in BA could reduce the levels of deconjugated and secondary bile acids, such as CDCA [18]. Therefore, we believe that it is logical to hypothesize that the GCDCA/CDCA ratio is an effective biomarker for increasing the diagnostic accuracy in BA patients because of the bile acid metabolism pathways.

In addition to bile acid, γ-GT and stool color have been used for the identification of BA in many previous studies [5, 8]. γ-GT levels were higher in infants with BA than in non-BA controls in our study, consistent with the results of other reports [5, 26]. γ-GT or stool color did not show good diagnostic ability in our study; however, the novel and most relevant finding of our study was that a combination of γ-GT, clay stool, and GCDCA/CDCA ratio overall improved the diagnostic performance of the tests. Besides, in our study, 29 (85.3%) BA patients had a positive HBS, which was defined as the absence of the radiotracer in the intestines for up to 24 h. However, 3 (14.7%) cases of BA showed negative HBS results, which means the radiotracer could be seen in the intestines for up to 24 h. Since BA is a progressive inflammatory cholangiopathy, and only 20% of BA patients showed complete fibroinflammatory obliteration [27]. We assumed that in those patients, the bile ducts were partially occluded by fibrosis. Presumably, the isotopes could pass through the slit-like lumen and transit into the duodenum in a few patients, which produced false-negative results, as demonstrated in this study. Also, according to a previous study, HBS has a high (98.7%) sensitivity but low (37–74%) specificity for BA diagnosis, with an overall diagnostic accuracy of 67% for BA [28]. A positive finding could also be found in severe intrahepatic cholestasis, such as CMV hepatitis, which reflects obstruction in the intrahepatic bile ducts affecting bile excretion in the intestine. HBS in the current study had a specificity of 56.3%, thus it was not selected by the multivariate logistic regression analysis, which might be due to its low specificity.

Several other models or scoring systems have been reported recently [17, 29]. El-Guindi et al. designed and validated a diagnostic score for BA with high sensitivity and specificity [29]. Nevertheless, the score included histopathological evaluation of liver biopsy. Generally, parents were unwilling to accept liver biopsy because of its cost and associated risks. By contrast, our scoring system could be easily and simply evaluated without invasive interventions. Moreover, the positive finding of our score could help guide the diagnostic assessment and could be a reference for the timing of intraoperative cholangiography.

Nevertheless, this study has some limitations. First, this study excluded some causes of infantile cholestasis, such as neonatal intrahepatic cholestasis caused by citrin deficiency, which might affect bile acid metabolism and had a different bile acid profile compared to those of other non-BA cholestasis [12]. Including metabolic diseases would affect the comparison between BA and non-BA groups. Furthermore, such diseases could be distinguished from BA by detecting amino acid profiles and genetic test. Second, bile acid detection has not been routinely used worldwide. Normal values for laboratory tests can vary from one laboratory to another. For better use of bile acid profiles, we converted our data into MoM values instead of using the actual measures. Third, because the differential of BA varies among populations of different ethnicities, the usefulness of the developed scoring system is limited to the Chinese population and validation in other ethnicities is required. Forth, though MoM is useful for evaluation when valuables depend on the instruments or reagents used, in general practice, each normal median value should be determined by testing those concentrations in normal control group. Therefore, calculating MoM is cumbersome in general clinical practices at present. However, since there remains no uniform measurement of bile acid concentration, we consider the MoM values is more reliable in current clinical practice. Also, we believe that with more application of this method in clinical practice, we could obtain a more feasible value to optimize our scoring system. Last, the rate of triangular cord sign was 8.8% in patients with biliary atresia in our deviation cohort, however, in the validation cohort, 9 of 40 (22.5%) cases of BA showed a positive find of TC sign. We assumed that the ultrasound findings depended on the experience of the radiologist, which made it inconsistent for use in the scoring system. However, we supposed that with the improvement of ultrasound technology and the experience of radiologists, the TC sign might be added to diagnosis scoring and may improve the accuracy of BA diagnosis.

## Conclusions

In conclusion, our study derived and validated a three-variable scoring system that provide a noninvasive diagnostic method for differentiating BA from cholestasis in infants. Using this score system, patients may potentially benefit from the timely indication for intraoperative cholangiography and would avoid unnecessary invasive procedures.

## Supplementary information

**Additional file 1: Table S1.** Bile acid assay of derivation cohort and normal controls by multiples of median value of normal control.

**Additional file 2: Table S2.** Point and corresponding risk estimation by risk category for BA in the derivation set.

**Additional file 3: Table S3.** Performance of the 3-measure score system in individual infants compared to the final confirmed diagnosis in the validation cohort (*n* = 75).

## Data Availability

The datasets used during the current study are available from the corresponding author on reasonable request.

## Abbreviations

BA: Biliary atresia
IOC: Intraoperative cholangiography
γ-GT: Gamma glutamyl transpeptidase
LC-MS/MS: Liquid chromatography tandem mass spectrometry
HBS: Hepatobiliary scintigraphy
US: Ultrasonography
IBAs: Individual bile acids
CA: Cholic acid
CDCA: Chenodeoxycholic acid
GCA: Glycocholic acid
GCDCA: Glycochenodeoxycholic acid
TCA: Taurocholic acid
TCDCA: Taurochenodeoxycholic acid
ROC: Receiver-operating characteristic
AUC: Area under receiver operating characteristic curve
MoM: Multiple of the median
