# Supplementary material for: Development and validation of bile acid profile-based scoring system for identification of biliary atresia: a prospective study
Source: BMC Pediatr. 2020 May 27;20:255. doi: 10.1186/s12887-020-02169-8 (PMC7251733; doi:10.1186/s12887-020-02169-8)
Supplement: Supplementary file 1 — Additional file 1: Table S1. Bile acid assay of derivation cohort and normal controls by multiples of median value of normal control. [file 12887_2020_2169_MOESM1_ESM.doc]

**Supplemental Table S1. Bile acid assay of derivation cohort and normal controls by multiples of median value of normal control.**

|  | **BA (**n=34) | **Non-BA (**n=32) | **Control (**n=37) | ***P***^a^ |
| --- | --- | --- | --- | --- |
| CA MoM | 0.1 (0.1, 0.1)^c^ | 0.1 (0.1, 0.2)^d^ | 1.0 (0.6, 1.8) | ***<0.001*** |
| CDCA MoM | 0.1 (0.1, 0.1)^c^ | 0.1 (0.1, 0.2)^d^ | 1.0 (0.5, 1.5) | ***<0.001*** |
| GDCA MoM | 2.7 (2.0, 3.9)^b,c^ | 1.0 (1.0, 3.0)^d^ | 1.0 (1.0, 1.0) | ***<0.001*** |
| GCA MoM | 10.0 (4.9, 14.2^)b,c^ | 3.7 (1.6, 7.2)^d^ | 1.0 (0.5, 2.2) | ***<0.001*** |
| GCDCA MoM | 4.5 (3.1, 9.3)^b,c^ | 2.2 (1.4, 4.3)^d^ | 1.0 (0.7, 1.6) | ***<0.001*** |
| TCA MoM | 14.3 (8.3, 22.4)^c^ | 10.6 (7.1, 17.7)^d^ | 1.0 (0.4, 2.9) | ***<0.001*** |
| TCDCA MoM | 10.5 (5.3, 17.5)^c^ | 8.4 (5.4, 12.0)^d^ | 1.0 (0.5, 2.4) | ***<0.001*** |
| GCDCA/CDCA MoM | 52.7 (30.3, 99)^b,c^ | 20.5 (7.7, 45.8)^d^ | 1.0 (0.5, 1.6) | ***<0.001*** |

Data are presented as median (IQR)

a P value: data were analyzed using Kruskal-Wallis test

b Paired comparisons with significance (p<0.017), using Mann-Whitney test between BA vs Non-BA

c Paired comparisons with significance (p<0.017), using Mann-Whitney test between BA vs Control

d Paired comparisons with significance (p<0.017), using Mann-Whitney test between Non-BA vs Control

MoM, multiple of median. CA, cholic acid; CDCA, chenodeoxycholic acid; GDCA, glycodeoxycholic acid; GCA, glycocholic acid; GCDCA, glycochenodeoxycholic acid; TCA, taurocholic acid; TCDCA, taurochenodeoxycholic acid
