# Supplementary material for: Development and validation of bile acid profile-based scoring system for identification of biliary atresia: a prospective study
Source: BMC Pediatr. 2020 May 27;20:255. doi: 10.1186/s12887-020-02169-8 (PMC7251733; doi:10.1186/s12887-020-02169-8)
Supplement: Supplementary file 2 — Additional file 2: Table S2. Point and corresponding risk estimation by risk category for BA in the derivation set. [file 12887_2020_2169_MOESM2_ESM.doc]

**Supplemental Table S2: Point and corresponding risk** **estimation by risk category for BA in the derivation set.**

| **BA risk category** | **Total point** | **Estimate of risk** |
| --- | --- | --- |
| **Low risk** | 0 | 0.074 |
|  | 5 | 0.150 |
|  | 10 | 0.281 |
|  | 15 | 0.465 |
| **High risk** | 20 | 0.659 |
|  | 25 | 0.811 |
|  | 30 | 0.905 |
|  | 35 | 0.955 |
|  | 40 | 0.979 |
|  | 41 | 0.982 |

BA: biliary atresia

Low risk: total point ≤15

High risk: total point >15
