# Supplementary material for: Development and validation of bile acid profile-based scoring system for identification of biliary atresia: a prospective study
Source: BMC Pediatr. 2020 May 27;20:255. doi: 10.1186/s12887-020-02169-8 (PMC7251733; doi:10.1186/s12887-020-02169-8)
Supplement: Supplementary file 3 — Additional file 3: Table S3. Performance of the 3-measure score system in individual infants compared to the final confirmed diagnosis in the validation cohort (n = 75). [file 12887_2020_2169_MOESM3_ESM.docx]

**Supplemental Table S3. Performance of the 3-measure score system in individual infants compared to the final confirmed diagnosis in the validation cohort (n=75).**

| **ID** | **Point** | **Diagnosis by the 3-measure model** | **Actual diagnosis** | **Final confirmed diagnosis** | **ID** | **Point** | **Diagnosis by the 3-measure model** | **Actual diagnosis** | **Final confirmed diagnosis** |
| --- | --- | --- | --- | --- | --- | --- | --- | --- | --- |
| **1** | 6 | non-BA | non-BA | Correct | **39** | 20 | BA | BA | Correct |
| **2** | 5 | non-BA | non-BA | Correct | **40** | 15 | non-BA | BA | **False** |
| **3** | 0 | non-BA | non-BA | Correct | **41** | 36 | BA | BA | Correct |
| **4** | 5 | non-BA | non-BA | Correct | **42** | 30 | BA | BA | Correct |
| **5** | 8 | non-BA | non-BA | Correct | **43** | 15 | non-BA | BA | **False** |
| **6** | 5 | non-BA | non-BA | Correct | **44** | 36 | BA | BA | Correct |
| **7** | 5 | non-BA | non-BA | Correct | **45** | 30 | BA | BA | Correct |
| **8** | 26 | BA | BA | Correct | **46** | 35 | BA | BA | Correct |
| **9** | 8 | non-BA | non-BA | Correct | **47** | 33 | BA | BA | Correct |
| **10** | 5 | non-BA | non-BA | Correct | **48** | 33 | BA | BA | Correct |
| **11** | 34 | BA | BA | Correct | **49** | 31 | BA | BA | Correct |
| **12** | 41 | BA | BA | Correct | **50** | 33 | BA | BA | Correct |
| **13** | 10 | non-BA | non-BA | Correct | **51** | 36 | BA | BA | Correct |
| **14** | 5 | non-BA | non-BA | Correct | **52** | 31 | BA | BA | Correct |
| **15** | 10 | non-BA | non-BA | Correct | **53** | 30 | BA | BA | Correct |
| **16** | 9 | non-BA | non-BA | Correct | **54** | 33 | BA | BA | Correct |
| **17** | 25 | BA | BA | Correct | **55** | 30 | BA | BA | Correct |
| **18** | 10 | non-BA | non-BA | Correct | **56** | 36 | BA | BA | Correct |
| **19** | 19 | BA | BA | Correct | **57** | 35 | BA | BA | Correct |
| **20** | 8 | non-BA | BA | **False** | **58** | 36 | BA | BA | Correct |
| **21** | 19 | BA | BA | Correct | **59** | 16 | BA | non-BA | **False** |
| **22** | 6 | non-BA | BA | **False** | **60** | 5 | non-BA | non-BA | Correct |
| **23** | 5 | non-BA | non-BA | Correct | **61** | 15 | non-BA | non-BA | Correct |
| **24** | 41 | BA | BA | Correct | **62** | 30 | BA | non-BA | **False** |
| **25** | 6 | non-BA | non-BA | Correct | **63** | 33 | BA | BA | Correct |
| **26** | 36 | BA | BA | Correct | **64** | 30 | BA | non-BA | **False** |
| **27** | 31 | BA | BA | Correct | **65** | 5 | non-BA | non-BA | Correct |
| **28** | 35 | BA | BA | Correct | **66** | 30 | BA | non-BA | **False** |
| **29** | 31 | BA | BA | Correct | **67** | 9 | non-BA | non-BA | Correct |
| **30** | 15 | non-BA | non-BA | Correct | **68** | 6 | non-BA | non-BA | Correct |
| **31** | 5 | non-BA | non-BA | Correct | **69** | 5 | non-BA | non-BA | Correct |
| **32** | 34 | BA | BA | Correct | **70** | 31 | BA | non-BA | **False** |
| **33** | 34 | BA | BA | Correct | **71** | 10 | non-BA | non-BA | Correct |
| **34** | 18 | BA | BA | Correct | **72** | 0 | non-BA | non-BA | Correct |
| **35** | 25 | BA | non-BA | **False** | **73** | 15 | non-BA | non-BA | Correct |
| **36** | 33 | BA | BA | Correct | **74** | 16 | BA | non-BA | **False** |
| **37** | 31 | BA | BA | Correct | **75** | 5 | non-BA | non-BA | Correct |
| 38 | 31 | BA | BA | Correct |  |  |  |  |  |

BA: biliary atresia
